# Supplementary material for: Genetic Background and Molecular Mechanisms of Juvenile Idiopathic Arthritis
Source: Int J Mol Sci. 2023 Jan 17;24(3):1846. doi: 10.3390/ijms24031846 (PMC9916312; doi:10.3390/ijms24031846)
Supplement: Supplementary file 1 [file ijms-24-01846-s001.zip › ijms-2157698-supplementary.pdf]

Table S1. Non-HLA loci with a reported susceptibility for various JIA subtypes [81–84,87,90,102,104,105]

Gene	Single nucleotide polymorphisms (SNP)	Minor allele frequency (MAF)	JIA subtypes	Reference (PMID)
ADGRL2	rs2066363	0.34	-	26301688
IL6	rs7808122	0.44	oJIA, RF – pJIA	23603761
	rs6946509	0.45	oJIA, RF – pJIA	23603761
IL6R	rs72698115	0.1	oJIA, RF – pJIA	23603761
	rs11265608	0.1	oJIA, RF – pJIA	23603761
STAT4	rs10174238	0.23	oJIA, RF – pJIA	23603761
	rs11889341	-	-	33106285
IL21-AS1	rs62324212	0.42	-	26301688
IL2-IL21	rs1479924	0.71	oJIA, RF – pJIA<br>RF + pJIA	23603761
ANKDR55<br>ANKRD55	rs10213692	0.75	oJIA, RF – pJIA	23603761
	rs7731626	0.39	-	26301688
	rs71624119	0.25	oJIA, RF – pJIA	23603761
LNPEP	rs27293	0.44	oJIA, RF – pJIA	23603761
	rs27290	0.44	oJIA, RF – pJIA	23603761
TENM3-AC114798.1	rs7660520	0.26	-	26301688
AC116366.3, C5orf56/IRF1	rs6894249	0.39-0.61	oJIA, RF – pJIA	23603761
	rs4705862	0.44	oJIA, RF – pJIA	23603761
IL2RA	rs7909519	0.11-0.89	oJIA, RF – pJIA<br>RF + pJIA	23603761
	rs706778	0.41	-	26301688
IL2RB	rs2284033	0.44-0.56	oJIA, RF – pJIA	23603761
MTCYBP42-AC073072.2	rs6946509	0.45	oJIA, RF – pJIA	23603761
FAS	rs7069750	0.44	oJIA, RF – pJIA	23603761
LINC00993, ANKRD30A	rs7100025	0.34	-	26301688
PRR5L, AC087277.1	rs7127214	0.65	oJIA, RF – pJIA	23603761
ATXN2	rs7137828	0.49	oJIA, RF – pJIA<br>RF + pJIA	23603761
	rs3184504	0.49	oJIA, RF – pJIA<br>RF + pJIA	23603761
	rs4766578	-	-	33106285
ZFP36L1	rs3825568	0.46-0.56	oJIA, RF – pJIA	23603761
	rs12434551	0.47	oJIA, RF – pJIA	23603761
PTPN2	rs2847293	0.17-0.35	oJIA, RF – pJIA<br>RF + pJIA	23603761
	rs9960807	-	-	33106285
TYK2	rs34536443	0.95	oJIA, RF – pJIA<br>RF + pJIA	23603761
PTPN22	rs6679677	0.1	oJIA, RF – pJIA<br>RF + pJIA	23603761
RUNX1	rs8129030	0.37-0.63	oJIA, RF – pJIA<br>RF + pJIA	23603761
	rs9979383	0.37	oJIA, RF – pJIA<br>RF + pJIA	23603761

RUNX3	rs4648881	0.49	oJIA, RF – pJIA	23603761
UBE2L3	rs2266959	0.19	oJIA, RF – pJIA<br>RF + pJIA	23603761
JAK1	rs10889504	0.13	oJIA, RF – pJIA	28719732
PRR9_LOR	rs873234	0.37	oJIA, RF – pJIA	28719732
PTH1R	rs1138518	0.37	oJIA, RF – pJIA	28719732
ILDR1_CD86	rs111700762	0.06	oJIA, RF – pJIA	28719732
LINC00951	rs10807228	0.33	oJIA, RF – pJIA	28719732
AHI1_LINC00271	rs9321502	0.4	oJIA, RF – pJIA	28719732
HBP1	rs111865019	0.27	oJIA, RF – pJIA	28719732
WDFY4	rs1904603	0.25	oJIA, RF – pJIA	28719732
RNF215	rs5753109	0.28	oJIA, RF – pJIA	28719732
LTBR	rs2364480	0.25	oJIA, RF – pJIA	23603761
	rs10849448	0.24	oJIA, RF – pJIA	23603761
COG6	rs7993214	0.35	oJIA, RF – pJIA<br>RF + pJIA	23603761
	rs9532434	0.36	oJIA, RF – pJIA<br>RF + pJIA	23603761
Chr13q14	rs34132030	0.32	oJIA, RF – pJIA	23603761
CCR1/CCR3	rs79893749	0.15	oJIA, RF – pJIA	23603761
PRR5L	rs4755450	0.35	oJIA, RF – pJIA	23603761
	rs7127214	0.35	oJIA, RF – pJIA	23603761
PRM1/C1orf75	rs66718203	0.18	oJIA, RF – pJIA	23603761
	rs11074967	0.42	oJIA, RF – pJIA	23603761
C3orf1/CD80	rs11714843	0.18	oJIA, RF – pJIA	23603761
	rs4688013	0.19	oJIA, RF – pJIA	23603761
JAZF1	rs10280937	0.11	oJIA, RF – pJIA<br>RF + pJIA	23603761
	rs73300638	0.11	oJIA, RF – pJIA<br>RF + pJIA	23603761
AFF3/LONRF2	rs6740838	0.39	oJIA, RF – pJIA<br>RF + pJIA	23603761
	rs10194635	0.39	oJIA, RF – pJIA<br>RF + pJIA	23603761
CLN3/CCDC101	rs497523	-	-	33106285
PADI4	rs12742364	0.15	All JIA subtypes	34101054
PRDM1	rs182399829	0.33	All JIA subtypes	34101054
LTBP1	rs219158	0.41	All JIA subtypes	34101054
ATG5	rs73776556	0.32	All JIA subtypes	34101054
ELMO1	rs2111035	0.16	All JIA subtypes	34101054
HAS2	rs1564569	0.25	All JIA subtypes	34101054
PRKG1	rs2454539	0.41	All JIA subtypes	34101054
CABIN1	rs9624387	0.03	All JIA subtypes	34101054
NFIA	rs1436238830	0.38	oJIA	34101054
VTCN1	rs11586858	0.26	oJIA	34101054
CD200R1	rs62263666	0.35	oJIA	34101054
UBE2D1	rs141412077	0.03	oJIA	34101054
ELMO1	rs2111035	0.16	oJIA	34101054
SPRY2	rs2154130	0.43	oJIA	34101054
CLEC16A	rs11074991	0.29	oJIA	34101054
VAV1	rs808843	0.1	oJIA	34101054

MX1	rs28449257	0.32	oJIA	34101054
IL1RN	rs55663133	0.22	sJIA	29609200
AJAP1	rs72632736	-	sJIA	27927641
COL11A1	rs1823549	-	sJIA	27927641
HDAC9	rs1178121	-	sJIA	27927641
ENC1-LOC101929082	rs12517545	-	sJIA	27927641
ZBTB7C	rs79575701	-	sJIA	27927641
KLF17	rs114940806	-	sJIA	27927641
LOC101929446	rs1279094	-	sJIA	27927641
PRICKLE2	rs864089	-	sJIA	27927641
ZNF37BP-ZNF33B	rs481331	-	sJIA	27927641
ZNF521-SS18	rs8097070	-	sJIA	27927641
EIF3H-LINC00536	rs1527934	-	sJIA	27927641
WWOX-LSM3P5	rs78507369	-	sJIA	27927641
LOC101928737-JPH3	rs12445022	-	sJIA	27927641
FBLN7	rs112165031	-	sJIA	27927641
RPF2P2-PGAM4P2	rs6853094	-	sJIA	27927641
LOC101927573-SORCS1	rs73401585	-	sJIA	27927641
CYSLTR2	rs9595973	-	sJIA	27927641
TRIM58	rs9633402	-	sJIA	27927641
LOC101928516-COL12A1	rs62438583	-	sJIA	27927641
LOC257396-MOCS2	rs62359376	-	sJIA	27927641
LDB2-TAPT1-ZEB2P1	rs1501138	-	sJIA	27927641
LINC01020- LOC101929176	rs7712113	-	sJIA	27927641
RIN3-LGMN	rs1885747	-	sJIA	27927641
MTHFSD-FOXL1-FOXC2	rs111580313	-	sJIA	27927641
